# Supplementary material for: Safety and efficacy of allylamines in the treatment of cutaneous and mucocutaneous leishmaniasis: A systematic review
Source: PLoS One. 2021 Apr 7;16(4):e0249628. doi: 10.1371/journal.pone.0249628 (PMC8026199; doi:10.1371/journal.pone.0249628)
Supplement: S1 Table — (DOCX) [file pone.0249628.s001.docx]

| **S1 Table. Characteristics of human case reports (n = 1) reporting treatment of cutaneous and mucocutaneous leishmaniasis with terbinafine** | | | | | | | | | |  |  |  |
| --- | --- | --- | --- | --- | --- | --- | --- | --- | --- | --- | --- | --- |
| **First Author** | **Year** | ***Leishmania* species studied** | **TL type** | **Reason to start with terbinafine** | **Presentation** | **Combination** | **Dose / day** | **Days treated** | **Effectivity** | **Adverse event rate** |  |  |
| Albanese [42] | 1994 | *tropica* | cutaneous | unknown | systemic | - | 500mg | 60 | yes | none |  |  |
| Gonzalez-Ruperez [43] | 1997 | unknown | cutaneous | initially diagnosed as tinea corporis | systemic | - | 250mg | 14 | yes | none |  |  |
| Vellin [45] | 2005 | *infantum* | mucocutaneous | unknown | systemic | Itraconazole 200mg/day | 500mg | 180 | yes | none |  |  |
| Scarisbrick [41] | 2006 | *tropica* | cutaneous | unknown | systemic | - | 250mg | ? | yes | none |  |  |
| Mawenzi [44] | 2018 | *unknown* | cutaneous | no other treatment available | systemic | Crotamiton + Sulphur | 250mg | 60 | yes | none |  |  |
