# Supplementary material for: Safety and efficacy of allylamines in the treatment of cutaneous and mucocutaneous leishmaniasis: A systematic review
Source: PLoS One. 2021 Apr 7;16(4):e0249628. doi: 10.1371/journal.pone.0249628 (PMC8026199; doi:10.1371/journal.pone.0249628)
Supplement: S1 File — (DOCX) [file pone.0249628.s002.docx]

MEDLINE

Database(s): **Ovid MEDLINE(R) and Epub Ahead of Print, In-Process & Other Non-Indexed Citations and Daily**1946 to May 22, 2020
Search Strategy: **2020-05-24**

| **#** | **Searches** | **Results** |
| --- | --- | --- |
| 1 | exp Leishmania/ or exp Leishmaniasis/ | 30937 |
| 2 | (Leishman* or antileishman* or Viannia).tw,kf,ot. | 35516 |
| 3 | ((L or V or promastigot* or amastigot*) adj2 (brazilien* or guyanen* or amazon* or mexican* or naiffi or lainsoni or panamensis or peruviana or shawi or aethiopica or major or killicki or tropica or infantum or chagasi)).tw,kf,ot. | 9705 |
| 4 | Montenegro*.tw,kf,ot. | 845 |
| **5** | **or/1-4 [Leishmania]** | **39358** |
| 6 | Allylamine/ | 714 |
| 7 | (butenafin* or SF-86-621 or terbinafin* or tanespimycin*).rn. [supplementary concepts] | 2624 |
| 8 | (allylamin* or allyl-amin* or propenylamin* or aminopropylen* or homoallylamin* or monoallylamin* or diallylamin* or triallylamin* or naphthalen*methylamin* or naphthylmethylamin* or ((naphthalen* or naphthyl) and methylamin*)).tw,kf. [allylamines] | 2873 |
| 9 | (terbinafin* or Lamisil or binasil or curasil or daskil or dermafin or exifine or interbi or labijin or lamifen or lapiderm or lespo or micoset or micosil or namuzol or SF-86-327 or SF-86327 or SF86-327 or SF86327 or sulmedin or terbifin* or terbinex or terbisil or terekol or terfine or termisil or SF-86-621 or SF-86621 or SF86-621 or SF86621).tw,kf. [terbinafine] | 2578 |
| 10 | (butenaf* or Mentax or KP-363 or KP363 or buticrem or butop or lotrimin or mentax or volley).tw,kf. [butenafine] | 1292 |
| 11 | (tanespimycin* or 17AAG* or 17-AAG* or CNF1010 or CNF-1010 or NSC-330507 or NSC330507 or 17allylamin* or IPI493 or IPI-493 or IPI504 or IPI-504 or retaspimycin* or demethoxygeldanamycin or 17demethoxygeldanamycin* or ((17demethoxy or demethoxy) adj3 geldanamycin*) or kos-953 or kos953 or nsc-330507 or nsc330507).tw,kf. [tanespimycine / 17-AAG] | 1219 |
| 12 | (naftifin* or naphthal*methylamin* or aw-105-843 or aw-105843 or aw105-843 or aw105843 or exoderil* or fetimin* or jia-mei or naftifungin or naftin* or naphthifungin* or sn-105-843 or sn-105843 or sn105-843 or sn105843 or suadian).tw,kf. [naftifine] | 197 |
| **13** | **or/6-12 [allylamines]** | **7947** |
| **14** | **5 and 13 [Leishmania & allylamines]** | **44** |
| **15** | **remove duplicates from 14 [Leishmania & allylamines]** | **43** |

EMBASE

Database(s): **Embase Classic+Embase**1947 to 2020 May 22
Search Strategy:

| # | Searches | Results |
| --- | --- | --- |
| 1 | exp leishmania/ or exp leishmaniasis/ or experimental cutaneous leishmaniasis/ or leishmanin skin test/ or leishmaniasis rapid test/ or antileishmanial agent/ or (antileishmanial activity or tegumentary leishmaniasis).dj. | 44469 |
| 2 | (Leishman* or antileishman* or Viannia).tw,kw,ot. | 41637 |
| 3 | ((L or V or promastigot* or amastigot*) adj2 (brazilien* or guyanen* or amazon* or mexican* or naiffi or lainsoni or panamensis or peruviana or shawi or aethiopica or major or killicki or tropica or infantum or chagasi)).tw,kw,ot. | 11280 |
| 4 | Montenegro*.tw,kw,ot. | 1221 |
| **5** | **or/1-4 [LEISHMANIA]** | **50426** |
| 6 | allylamine/ or allylamine derivative/ | 1493 |
| 7 | (allylamin* or allyl-amin* or propenylamin* or aminopropylen* or homoallylamin* or monoallylamin* or diallylamin* or triallylamin* or naphthalen*methylamin* or naphthylmethylamin* or ((naphthalen* or naphthyl) and methylamin*)).tw,kw. [allylamines] | 3566 |
| 8 | terbinafine/ | 7655 |
| 9 | (terbinafin* or Lamisil or binasil or curasil or daskil or dermafin or exifine or interbi or labijin or lamifen or lapiderm or lespo or micoset or micosil or namuzol or SF-86-327 or SF-86327 or SF86-327 or SF86327 or sulmedin or terbifin* or terbinex or terbisil or terekol or terfine or termisil or SF-86-621 or SF-86621 or SF86-621 or SF86621).tw,kw. [terbinafine] | 4244 |
| 10 | butenafine/ | 1216 |
| 11 | (butenaf* or Mentax or KP-363 or KP363 or buticrem or butop or lotrimin or mentax or volley).tw,kw. | 2220 |
| 12 | tanespimycin/ | 2206 |
| 13 | (tanespimycin* or 17AAG* or 17-AAG* or CNF1010 or CNF-1010 or NSC-330507 or NSC330507 or 17allylamin* or IPI493 or IPI-493 or IPI504 or IPI-504 or retaspimycin* or demethoxygeldanamycin or 17demethoxygeldanamycin* or ((17demethoxy or demethoxy) adj3 geldanamycin*) or kos-953 or kos953 or nsc-330507 or nsc330507).tw,kw. | 2269 |
| 14 | naftifine/ | 694 |
| 15 | (naftifin* or naphthal*methylamin* or aw-105-843 or aw-105843 or aw105-843 or aw105843 or exoderil* or fetimin* or jia-mei or naftifungin or naftin* or naphthifungin* or sn-105-843 or sn-105843 or sn105-843 or sn105843 or suadian).tw,kw. [naftifine] | 368 |
| **16** | **or/6-15 [ALLYLAMINES]** | **16764** |
| **17** | **5 and 16 [LEISHMANIA & ALLYLAMINES]** | **153** |
| **18** | **remove duplicates from 17 [LEISHMANIA & ALLYLAMINES - DEDUPLICATED]** | **152** |
| **19** | **18 not medline.cr. [LEISHMANIA & ALLYLAMINES - DEDUPLICATED - EMBASE ONLY]** | **146** |

CENTRAL

**2020-05-24**

| ID | Search | 2020-05-24 |
| --- | --- | --- |
| #1 | (Leishman* or antileishman* or Viannia):ti,ab,kw | 828 |
| #2 | (((L or V or promastigot* or amastigot*) near/2 (brazilien* or guyanen* or amazon* or mexican* or naiffi or lainsoni or panamensis or peruviana or shawi or aethiopica or killicki or tropica or infantum or chagasi)) or (L NEXT major)):ti,ab,kw | 120 |
| #3 | Montenegro*:ti,ab,kw | 38 |
| **#4** | **{or #1-#3}** | **868** |
| #5 | (allylamin* or (allyl NEXT amin*) or propenylamin* or aminopropylen* or homoallylamin* or monoallylamin* or diallylamin* or triallylamin* or naphthalen*methylamin* or naphthylmethylamin* or ((naphthalen* or naphthyl) and methylamin*)):ti,ab,kw | 160 |
| #6 | (terbinafin* or Lamisil or binasil or curasil or daskil or dermafin or exifine or interbi or labijin or lamifen or lapiderm or lespo or micoset or micosil or namuzol or (SF NEXT 86 NEXT 327) or (SF NEXT 86327) or (SF86 NEXT 327) or SF86327 or sulmedin or terbifin* or terbinex or terbisil or terekol or terfine or termisil or (SF NEXT 86 NEXT 621) or (SF NEXT 86621) or (SF86 NEXT 621) or SF86621):ti,ab,kw | 533 |
| #7 | (butenaf* or Mentax or (KP NEXT 363) or KP363 or buticrem or butop or lotrimin or mentax or volley):ti,ab,kw | 82 |
| #8 | (tanespimycin* or 17AAG* or (17 NEXT AAG*) or CNF1010 or (CNF NEXT 1010) or (NSC NEXT 330507) or NSC330507 or 17allylamin* or IPI493 or (IPI NEXT 493) or IPI504 or (IPI NEXT 504) or retaspimycin* or demethoxygeldanamycin or 17demethoxygeldanamycin* or ((17demethoxy or demethoxy) near/3 geldanamycin*) or (kos NEXT 953) or kos953 or (nsc NEXT 330507) or nsc330507):ti,ab,kw | 19 |
| #9 | (naftifin* or naphthal*methylamin* or (aw NEXT 105 NEXT 843) or (aw NEXT 105843) or (aw105 NEXT 843) or aw105843 or exoderil* or fetimin* or (jia NEXT mei) or naftifungin or naftin* or naphthifungin* or (sn NEXT 105 NEXT 843) or (sn NEXT 105843) or (sn105 NEXT 843) or sn105843 or suadian):ti,ab,kw | 83 |
| **#10** | **{or #5-#9}** | **785** |
| **#11** | **#4 and #10** | **4** |

Global Health Library **2020-05-24**

(Leishman* or antileishman* or Viannia) **AND** (allylamin* or allyl-amin* or propenylamin* or aminopropylen* or homoallylamin* or monoallylamin* or diallylamin* or triallylamin* or naphthalen*methylamin* or naphthylmethylamin* or ((naphthalen* or naphthyl) and methylamin*) or terbinafin* or Lamisil or binasil or curasil or daskil or dermafin or exifine or interbi or labijin or lamifen or lapiderm or lespo or micoset or micosil or namuzol or SF-86-327 or SF-86327 or SF86-327 or SF86327 or sulmedin or terbifin* or terbinex or terbisil or terekol or terfine or termisil or SF-86-621 or SF-86621 or SF86-621 or SF86621 or butenaf* or Mentax or KP-363 or KP363 or buticrem or butop or lotrimin or mentax or volley or tanespimycin* or 17AAG* or 17-AAG* or CNF1010 or CNF-1010 or NSC-330507 or NSC330507 or 17allylamin* or IPI493 or IPI-493 or IPI504 or IPI-504 or retaspimycin* or demethoxygeldanamycin or 17demethoxygeldanamycin* or kos-953 or kos953 or nsc-330507 or nsc330507 or naftifin* or naphthal*methylamin* or aw-105-843 or aw-105843 or aw105-843 or aw105843 or exoderil* or fetimin* or jia-mei or naftifungin or naftin* or naphthifungin* or sn-105-843 or sn-105843 or sn105-843 or sn105843 or suadian)

**8 hits**

Web of Science

**2020-05-24**

TS=(Leishman* or antileishman* or Viannia) AND TS=(allylamin* or allyl-amin* or propenylamin* or aminopropylen* or homoallylamin* or monoallylamin* or diallylamin* or triallylamin* or naphthalen*methylamin* or naphthylmethylamin* or ((naphthalen* or naphthyl) and methylamin*) or terbinafin* or Lamisil or binasil or curasil or daskil or dermafin or exifine or interbi or labijin or lamifen or lapiderm or lespo or micoset or micosil or namuzol or SF-86-327 or SF-86327 or SF86-327 or SF86327 or sulmedin or terbifin* or terbinex or terbisil or terekol or terfine or termisil or SF-86-621 or SF-86621 or SF86-621 or SF86621 or butenaf* or Mentax or KP-363 or KP363 or buticrem or butop or lotrimin or mentax or volley or tanespimycin* or 17AAG* or 17-AAG* or CNF1010 or CNF-1010 or NSC-330507 or NSC330507 or 17allylamin* or IPI493 or IPI-493 or IPI504 or IPI-504 or retaspimycin* or demethoxygeldanamycin or 17demethoxygeldanamycin* or ((17demethoxy or demethoxy) NEAR/3 geldanamycin*) or kos-953 or kos953 or nsc-330507 or nsc330507 or naftifin* or naphthal*methylamin* or aw-105-843 or aw-105843 or aw105-843 or aw105843 or exoderil* or fetimin* or jia-mei or naftifungin or naftin* or naphthifungin* or sn-105-843 or sn-105843 or sn105-843 or sn105843 or suadian)

**Indexes=SCI-EXPANDED, SSCI, A&HCI, ESCI Timespan=All years (all documents)**

**50 hits all databases**

Google scholar

2020-05-24

"Leishmania"|"leishmaniasis"|"antileishmania"|"Viannia" "allylamine"|"allylamines"|"allyl-amine"|"allyl-amines"|"terbinafine"|"Lamisil"|"SF 86–327"|"butenafine"|"tanespimycin"|"17AAG"|"17-AAG"|"17allylamine"|"naftifine"

**First 150 records, highest ranked (via Harzing’s Publish and Perish 6)**

Clinicaltrials.gov

2020-05-24

No Studies found for: **(allylamine OR allyl-amine OR terbinafine OR Lamisil OR 86–327 OR butenafine OR tanespimycine OR 17AAG OR 17-AAG OR 17allylamine OR naftifine) AND leishmaniasis**

International Clinical Trials Registry Platfrom (WHO)

| <http://apps.who.int/trialsearch/default.aspx>  **2020-05-24**  2 records for 2 trials found for: leishman* AND allylamin* OR leishman* AND allyl-amin* OR leishman* AND terbinafin* OR leishman* AND Lamisil OR leishman* AND 86–327 OR leishman* AND butenafin* OR leishman* AND tanespimycin* OR leishman* AND 17AAG OR leishman* AND 17-AAG OR leishman* AND 17allylamin* OR leishman* AND naftifine  **2 records** in the Australian New Zealand Clinical Trials registry ANZCTR |  |
| --- | --- |

To import records downloaded from the WHO International Clinical Trials Registry Platform (ICTRP). These can only be downloaded as a .xml file. Import the .xml file using this filter with No Text Translation.
