# Supplementary material for: Safety and efficacy of allylamines in the treatment of cutaneous and mucocutaneous leishmaniasis: A systematic review
Source: PLoS One. 2021 Apr 7;16(4):e0249628. doi: 10.1371/journal.pone.0249628 (PMC8026199; doi:10.1371/journal.pone.0249628)
Supplement: S2 File — (DOCX) [file pone.0249628.s003.docx]

| **Section/topic** | **#** | **Checklist item** | **Reported on page #** |
| --- | --- | --- | --- |
| **TITLE** | | |  |
| Title | 1 | Identify the report as a systematic review, meta-analysis, or both.  *Safety and efficacy of allylamines in the treatment of cutaneous and mucocutaneous leishmaniasis:*  *a systematic review* | 1 |
| **ABSTRACT** | | |  |
| Structured summary | 2 | Provide a structured summary including, as applicable:  background; *Cutaneous and mucocutaneous leishmaniasis affect a million people yearly*  objectives; *This review assesses efficacy and safety of allylamines for the treatment of cutaneous and mucocutaneous leishmaniasis*  data sources; *MEDLINE, EMBASE, the Cochrane Central Register of Controlled Trials, the Global Health Library, Web of Science, Google Scholar and clinical trial registers were searched*  study eligibility criteria, participants, and interventions; *All original human, animal, and in vitro studies concerning allylamines and cutaneous or mucocutaneous leishmaniasis were eligible for inclusion.*  study appraisal and synthesis methods; *A meta-analysis could not be performed due to the small number of studies, their heterogeneity, and low quality.*  results; *The search identified 312 publications*  limitations; *A meta-analysis could not be performed due to the small number of studies, their heterogeneity and low quality.*  conclusions and implications of key findings*; This systematic review shows that there is no evidence of efficacy of allylamine monotherapy against cutaneous and mucocutaneous leishmaniasis. Further trials of allylamines should be carefully considered as the outcomes of an adequately designed trial were disappointing and in vitro studies indicate minimal effective concentrations that are not achieved in the skin during standard doses.*  Systematic review registration numbe*r. preregistered in PROSPERO (CRD4201809068)* | 2,3 |
| **INTRODUCTION** | | |  |
| Rationale | 3 | Describe the rationale for the review in the context of what is already known.  *Depending on the infecting Leishmania species, multiple treatment options are available but pentavalent antimonials (e.g., sodium stibogluconate and meglumine antimoniate) are still the most frequently used. Yet, antimonial therapy is painful and requires multiple intralesional, intravenous, or intramuscular injections up to 30 days [*[*5*](#_ENREF_5)*,* [*6*](#_ENREF_6)*]. Miltefosine, the oral alternative for systemic CL and MCL therapy, is not widely available and very expensive, limiting its use in clinical practice* | 3 |
| Objectives | 4 | Provide an explicit statement of questions being addressed with reference to  Participants: *human, animal and in vitro*  interventions: *allylamines*  comparisons: *both placebo or alternative CL and ML treatments.*  Outcomes: *Cure rate in humans, change in lesion diameter in animals, promastigote and amastigote viability and growth, and adverse events served as outcome.*  and study design (PICOS): *All original human, animal and in vitro studies* | 3,4 |
| **METHODS** | | |  |
| Protocol and registration | 5 | Indicate if a review protocol exists, if and where it can be accessed (e.g., Web address), and, if available, provide registration information including registration number. <https://www.crd.york.ac.uk/prospero/display_record.php?RecordID=90687>  *PROSPERO (registration number CRD42018090687, 2018)* | 5 |
| Eligibility criteria | 6 | Specify study characteristics (e.g., PICOS, length of follow-up) and report characteristics (e.g., years considered, language, publication status) used as criteria for eligibility, giving rationale.  *All original human, animal, and in vitro studies* | 5 |
| Information sources | 7 | Describe all information sources (e.g., databases with dates of coverage, contact with study authors to identify additional studies) in the search and date last searched. *A medical information specialist (JL) searched the following electronic databases for studies on leishmaniasis and allylamines, using controlled terms and text words from inception to March, 4^th^ 2019: MEDLINE (OVID), EMBASE (OVID), the Cochrane Central Register of Controlled Trials (CENTRAL), The Global Health Library (including LILACS, IMEMR and WPRIM and SciELO), Web of Science, Google Scholar (1^st^ 150 hits) and the clinical trial registers ClinicalTrials.gov and WHO_ICTRP without language or date restrictions.*  *Reference lists and the citing articles of the identified relevant papers were cross-checked in Web of Science for additional relevant studies.* | 5 |
| Search | 8 | Present full electronic search strategy for at least one database, including any limits used, such that it could be repeated.  *The complete search strategies are presented in the S1 File.* | 5 |
| Study selection | 9 | State the process for selecting studies (i.e., screening, eligibility, included in systematic review, and, if applicable, included in the meta-analysis).  *JB and JvdE independently screened the identified studies using EndNote and resolved differences through discussion or consultation with a third reviewer (HS).* | 6 |
| Data collection process | 10 | Describe method of data extraction from reports (e.g., piloted forms, independently, in duplicate) and any processes for obtaining and confirming data from investigators.  *The following data from the eligible studies was entered in Excel*  *JB and JvdE extracted data in duplicate and resolved differences trough discussion* | 6 |
| Data items | 11 | List and define all variables for which data were sought (e.g., PICOS, funding sources) and any assumptions and simplifications made.  *The following data from all included studies were entered in Excel: study setting, study population, probable Leishmania species, allylamine studied and treatment combinations.* | 6 |
| Risk of bias in individual studies | 12 | Describe methods used for assessing risk of bias of individual studies (including specification of whether this was done at the study or outcome level), and how this information is to be used in any data synthesis.  *JB and JvdE independently assessed the quality of clinical trials, using the revised Cochrane collaborations tool for assessing risk of bias in randomized controlled trials and the Cochrane tool for non-randomised controlled trials. Animal studies were assessed with the SYRCLE´s risk of bias assessment tool.* | 6,7 |
| Summary measures | 13 | State the principal summary measures (e.g., risk ratio, difference in means).  *It was not possible to perform a meta analysis of the study outcomes* | 10 |
| Synthesis of results | 14 | Describe the methods of handling data and combining results of studies, if done, including measures of consistency (e.g., I^2^) for each meta-analysis.  *It was not possible to perform a meta analysis of the study outcomes* | 10 |

Page 1 of 2

| **Section/topic** | **#** | **Checklist item** | **Reported on page #** |
| --- | --- | --- | --- |
| Risk of bias across studies | 15 | Specify any assessment of risk of bias that may affect the cumulative evidence (e.g., publication bias, selective reporting within studies). *Meta analysis was not performed* | - |
| Additional analyses | 16 | Describe methods of additional analyses (e.g., sensitivity or subgroup analyses, meta-regression), if done, indicating which were pre-specified. *Meta analysis was not performed* | - |
| **RESULTS** | | |  |
| Study selection | 17 | Give numbers of studies screened, assessed for eligibility, and included in the review, with reasons for exclusions at each stage, ideally with a flow diagram.  *The literature search identified 312 unique studies of which 75 were included for full text assessment. After full text examination, 22 studies were included.*  *(Fig 1)* | 7 |
| Study characteristics | 18 | For each study, present characteristics for which data were extracted (e.g., study size, PICOS, follow-up period) and provide the citations.  *Characteristics of the included human trials (n = 3) [*[*25-27*](#_ENREF_25)*], mice studies (n = 2) [*[*28*](#_ENREF_28)*,* [*29*](#_ENREF_29)*], and amastigote and promastigote studies (n = 12) [*[*11*](#_ENREF_11)*,* [*30-40*](#_ENREF_30)*] are presented in Tables 1 and 2. The case reports (n = 5) [*[*41-45*](#_ENREF_41)*] are presented in S1 Table.* | 7 |
| Risk of bias within studies | 19 | Present data on risk of bias of each study and, if available, any outcome level assessment (see item 12).  *The 12 in vitro studies presented minor methodological risks of bias (Figs 2-5).* | 11 |
| Results of individual studies | 20 | For all outcomes considered (benefits or harms), present, for each study: (a) simple summary data for each intervention group (b) effect estimates and confidence intervals, ideally with a forest plot.  ***Table 3. Overview of clinical and in vitro Leishmania species specific results of terbinafine in cutaneous leishmaniasis*** | 12 |
| Synthesis of results | 21 | Present results of each meta-analysis done, including confidence intervals and measures of consistency.  *Meta analysis was not performed* | - |
| Risk of bias across studies | 22 | Present results of any assessment of risk of bias across studies (see Item 15).  *Meta analysis was not performed* | - |
| Additional analysis | 23 | Give results of additional analyses, if done (e.g., sensitivity or subgroup analyses, meta-regression [see Item 16]).  *Meta analysis was not performed* | - |
| **DISCUSSION** | | |  |
| Summary of evidence | 24 | Summarize the main findings including the strength of evidence for each main outcome; consider their relevance to key groups (e.g., healthcare providers, users, and policy makers).  This systematic review assesses efficacy and safety of allylamines for the treatment of CL and MCL. It comprises an exhaustive search of eight electronic databases and trial registers. It assesses the risk of bias of two randomised controlled trials, a non-controlled trial, two animal studies, and twelve in vitro studies and summarizes the available evidence including five case reports. Generally, the quality of evidence was low and human studies were done only in L. tropica. | 15 |
| Limitations | 25 | Discuss limitations at study and outcome level (e.g., risk of bias), and at review-level (e.g., incomplete retrieval of identified research, reporting bias).  Generally, the quality of evidence was low and human studies were done only in L. tropica. | 13,14 |
| Conclusions | 26 | Provide a general interpretation of the results in the context of other evidence, and implications for future research.  *Based on a systematic review of available literature we conclude that there is no evidence for the efficacy of allylamine monotherapy against CL and MCL.*  *However, the in vitro synergistic effects of allylamines combined with triazole drugs against amastigotes, warrant more investigation starting with high quality animal studies to define optimal doses and safety profiles and followed by well-designed trials in humans in case of positive findings.* | 15 |
| **FUNDING** | | |  |
| Funding | 27 | Describe sources of funding for the systematic review and other support (e.g., supply of data); role of funders for the systematic review.  *JB received a monthly volunteer allowance from the foundation Latin Link Nederland.*  *Funders are mentioned online at submission for PLOS ONE* | - |

*From:*  Moher D, Liberati A, Tetzlaff J, Altman DG, The PRISMA Group (2009). Preferred Reporting Items for Systematic Reviews and Meta-Analyses: The PRISMA Statement. PLoS Med 6(7): e1000097. doi:10.1371/journal.pmed1000097

For more information, visit: **www.prisma-statement.org**.

Page 2 of 2
