# Supplementary material for: Safety and efficacy of allylamines in the treatment of cutaneous and mucocutaneous leishmaniasis: A systematic review
Source: PLoS One. 2021 Apr 7;16(4):e0249628. doi: 10.1371/journal.pone.0249628 (PMC8026199; doi:10.1371/journal.pone.0249628)
Supplement: Attachment — Submitted filename: PONE-D-20-23096_reviewer_13oct.pdf [file pone.0249628.s004.pdf]

PLOS ONE

Safety and efficacy of allylamines in the treatment of tegumentary leishmaniasis: a systematic review

--Manuscript Draft--

Manuscript Number:	PONE-D-20-23096
Article Type:	Research Article
Full Title:	Safety and efficacy of allylamines in the treatment of tegumentary leishmaniasis: a systematic review
Short Title:	Allylamines for tegumentary leishmaniasis: a systematic review
Corresponding Author:	Jacob M Bezemer, MD<br>University of Amsterdam<br>Amsterdam, Noord Holland NETHERLANDS
Keywords:	systematic review; tegumentary leishmaniasis; cutaneous leishmaniasis; mucocutaneous leishmaniasis; allylamine; terbinafine; butenafine; naftifine; treatment
Abstract:	Background<br>Tegumentary Leishmaniasis (TL) affects a million people yearly. Infection with Leishmania parasites causing TL can result in skin ulcers or lethal mucosal disease. Current TL treatments have severe side effects. Allylamine drugs, like terbinafine, are considered to be safe, also during pregnancy and lactation. This review assesses efficacy and safety of allylamines for the treatment of TL.Methodology This systematic review followed the PRISMA statement for reporting and was preregistered in PROSPERO(CRD4201809068). MEDLINE, EMBASE, the Cochrane Central Register of Controlled Trials, the Global Health Library, Web of Science, Google Scholar and clinical trial registers were searched from inception to May, 24th, 2020. All original human, animal and in vitro studies, including studies comparing effects of allylamines with placebo or alternative treatments on cure, growth inhibition or adverse events, were eligible for inclusion. Risk of bias in human and animal studies was assessed with the Cochrane and SYRCLE´s tools respectively.FindingsThe search identified 312 publications, of which 22 were included in this systematic review. There were one uncontrolled and 2 randomised controlled trials. The only well-designed randomised controlled trial that compared the treatment efficacy of oral terbinafine versus intra muscular meglumine antimoniate in 80 Leismania tropica infected patients showed a non-significant lower cure rate for terbinafine vs meglumine antimoniate (38% vs 53%). Allylamines did not eliminate amastigotes at clinically relevant levels in in vitro studies. However, terbinafine combined with triazole drugs showed synergistic in vitro inhibition of promastigotes and amastigotes. A meta-analysis could not be performed due to the small number of studies, their heterogeneity and low quality.Conclusions<br>This systematic review shows that allylamine monotherapy is not effective in TL in clinical trials and in vitro models. However, the in vitro synergistic effects of allylamines combined with triazole drugs warrant further exploration.
Order of Authors:	Jacob M Bezemer, MD
	Jacob van der Ende
	Jacqueline Limpens
	Henry J C de Vries
	Henk D F H Schallig
Additional Information:	
**Question**	**Response**
**Financial Disclosure**<br><br>Enter a financial disclosure statement that describes the sources of funding for the	JB received a monthly volunteer allowance from Latin Link Nederland<br>http://www.latinlink-nederland.nl/<br>Latin Link had no role in study design, data collection and analysis, decision to publish, preparation of the manuscript

Powered by Editorial Manager® and ProduXion Manager® from Aries Systems Corporation

work included in this submission. Review the submission guidelines for detailed requirements. View published research articles from *PLOS ONE* for specific examples.<br><br>This statement is required for submission and **will appear in the published article** if the submission is accepted. Please make sure it is accurate.<br><br>**Unfunded studies**<br>Enter: *The author(s) received no specific funding for this work.*<br><br>**Funded studies**<br>Enter a statement with the following details:<br>• Initials of the authors who received each award<br>• Grant numbers awarded to each author<br>• The full name of each funder<br>• URL of each funder website<br>• Did the sponsors or funders play any role in the study design, data collection and analysis, decision to publish, or preparation of the manuscript?<br>• **NO** - Include this sentence at the end of your statement: *The funders had no role in study design, data collection and analysis, decision to publish, or preparation of the manuscript.*<br>• **YES** - Specify the role(s) played.<br><br>* typeset	
**Competing Interests**<br><br>Use the instructions below to enter a competing interest statement for this submission. On behalf of all authors, disclose any competing interests that could be perceived to bias this work—acknowledging all financial support and any other relevant financial or non-financial competing interests.<br><br>This statement **will appear in the published article** if the submission is accepted. Please make sure it is accurate. View published research articles from *PLOS ONE* for specific examples.	The authors have declared that no competing interests exist.

Powered by Editorial Manager® and ProduXion Manager® from Aries Systems Corporation

**NO authors have competing interests**<br><br>Enter: *The authors have declared that no competing interests exist.*<br><br>**Authors with competing interests**<br><br>Enter competing interest details beginning with this statement:<br><br>*I have read the journal's policy and the authors of this manuscript have the following competing interests: [insert competing interests here]*<br><br>* typeset	
**Ethics Statement**<br><br>Enter an ethics statement for this submission. This statement is required if the study involved:<br><br>• Human participants<br>• Human specimens or tissue<br>• Vertebrate animals or cephalopods<br>• Vertebrate embryos or tissues<br>• Field research<br><br>Write "N/A" if the submission does not require an ethics statement.<br><br>General guidance is provided below. Consult the submission guidelines for detailed instructions. **Make sure that all information entered here is included in the Methods section of the manuscript.**	N/A

Powered by Editorial Manager® and ProduXion Manager® from Aries Systems Corporation

**Format for specific study types**<br><br>**Human Subject Research (involving human participants and/or tissue)**<br>• Give the name of the institutional review board or ethics committee that approved the study<br>• Include the approval number and/or a statement indicating approval of this research<br>• Indicate the form of consent obtained (written/oral) or the reason that consent was not obtained (e.g. the data were analyzed anonymously)<br><br>**Animal Research (involving vertebrate animals, embryos or tissues)**<br>• Provide the name of the Institutional Animal Care and Use Committee (IACUC) or other relevant ethics board that reviewed the study protocol, and indicate whether they approved this research or granted a formal waiver of ethical approval<br>• Include an approval number if one was obtained<br>• If the study involved *non-human primates*, add *additional details* about animal welfare and steps taken to ameliorate suffering<br>• If anesthesia, euthanasia, or any kind of animal sacrifice is part of the study, include briefly which substances and/or methods were applied<br><br>**Field Research**<br><br>Include the following details if this study involves the collection of plant, animal, or other materials from a natural setting:<br>• Field permit number<br>• Name of the institution or relevant body that granted permission	
**Data Availability**<br><br>Authors are required to make all data underlying the findings described fully available, without restriction, and from the time of publication. PLOS allows rare exceptions to address legal and ethical concerns. See the PLOS Data Policy and FAQ for detailed information.	Yes - all data are fully available without restriction

A Data Availability Statement describing where the data can be found is required at submission. Your answers to this question constitute the Data Availability Statement and **will be published in the article**, if accepted.<br><br>**Important:** Stating 'data available on request from the author' is not sufficient. If your data are only available upon request, select 'No' for the first question and explain your exceptional situation in the text box.<br><br>Do the authors confirm that all data underlying the findings described in their manuscript are fully available without restriction?	
**Describe where the data may be found in full sentences. If you are copying our sample text, replace any instances of XXX with the appropriate details.**<br><br>• If the data are **held or will be held in a public repository**, include URLs, accession numbers or DOIs. If this information will only be available after acceptance, indicate this by ticking the box below. For example: *All XXX files are available from the XXX database (accession number(s) XXX, XXX.).*<br>• If the data are all contained **within the manuscript and/or Supporting Information files**, enter the following: *All relevant data are within the manuscript and its Supporting Information files.*<br>• If neither of these applies but you are able to provide **details of access elsewhere**, with or without limitations, please do so. For example:<br><br>*Data cannot be shared publicly because of [XXX]. Data are available from the XXX Institutional Data Access / Ethics Committee (contact via XXX) for researchers who meet the criteria for access to confidential data.*<br><br>*The data underlying the results presented in the study are available from (include the name of the third party*	All relevant data are within the manuscript and its Supporting Information files.

and contact information or URL).<br>• This text is appropriate if the data are owned by a third party and authors do not have permission to share the data.<br><br>* typeset	
Additional data availability information:	

Manuscript

Click here to access/download;Manuscript;PLOS one manuscript.docx

Safety and efficacy of allylamines in the treatment of tegumentary leishmaniasis: a systematic review

Allylamines for tegumentary leishmaniasis: a systematic review

Jacob M Bezemer[1,2*], Jacob van der Ende[3], Jacqueline Limpens[4], Henry J C de Vries[5], Henk D F H Schallig[1]

[1]Experimental Parasitology Unit, Department of Medical Microbiology, Amsterdam UMC, University of Amsterdam, Amsterdam, the Netherlands

[2] Fundación Misión Cristiana de Salud, Shell, Pastaza, Ecuador

[3] Fundación Quina Care Ecuador, Puerto el Carmen de Putumayo, Sucumbíos, Ecuador

[4] Medical Library, Amsterdam UMC, University of Amsterdam, Amsterdam, the Netherlands.

[5] Department of Dermatology, Amsterdam Institute for infection and immunity, Amsterdam UMC, University of Amsterdam, Amsterdam, the Netherlands

[*] Corresponding author

E-mail: j.m.bezemer@amsterdamumc.nl (JB)

Abstract

Background

Tegumentary Leishmaniasis (TL) affects a million people yearly. Infection with *Leishmania* parasites causing TL can result in skin ulcers or lethal mucosal disease. Current TL treatments have severe side effects. Allylamine drugs, like terbinafine, are considered to be safe, also during pregnancy and lactation. This review assesses efficacy and safety of allylamines for the treatment of TL.

Methodology

This systematic review followed the PRISMA statement for reporting and was preregistered in PROSPERO(CRD4201809068). MEDLINE, EMBASE, the Cochrane Central Register of Controlled Trials, the Global Health Library, Web of Science, Google Scholar and clinical trial registers were searched from inception to May, 24th, 2020. All original human, animal and *in vitro* studies, including studies comparing effects of allylamines with placebo or alternative treatments on cure, growth inhibition or adverse events, were eligible for inclusion. Risk of bias in human and animal studies was assessed with the Cochrane and SYRCLE´s tools respectively.

Findings

The search identified 312 publications, of which 22 were included in this systematic review. There were one uncontrolled and 2 randomised controlled trials. The only well-designed randomised controlled trial that compared the treatment efficacy of oral terbinafine versus intra muscular meglumine antimoniate in 80 *Leismania tropica* infected patients showed a non-significant lower

cure rate for terbinafine vs meglumine antimoniate (38% vs 53%). Allylamines did not eliminate amastigotes at clinically relevant levels in *in vitro* studies. However, terbinafine combined with triazole drugs showed synergistic *in vitro* inhibition of promastigotes and amastigotes. A meta-analysis could not be performed due to the small number of studies, their heterogeneity and low quality.

Conclusions

This systematic review shows that allylamine monotherapy is not effective in TL in clinical trials and in vitro models. However, the *in vitro* synergistic effects of allylamines combined with triazole drugs warrant further exploration.

Introduction

Background

Tegumentary Leishmaniasis (TL), classified by the World Health Organization (WHO) as an emerging neglected disease, affects more than one million people yearly [1, 2]. TL manifests as skin ulcers or deadly mucosal disease (mucocutaneous leishmaniasis [MCL] characterized by destructive inflammation of the nose, mouth and larynx) [3]. At least twenty different *Leishmania* parasite species can cause TL and each species responds differently to treatment [4]. Pentavalent antimonials (e.g. pentamidine and meglumine) are most frequently used in the treatment of TL, but they have to be administered intravenously or intramuscularly up to 30 days. Moreover, pentavalent antimonials can result in hepatotoxicity, renal insufficiency, pancreatitis, cardiac arrest and other serious side effects and there is no safe alternative drug for use in pregnant or lactating women [5]. Furthermore, poor treatment responses exist for TL drugs [6]. Consequently, there is a pressing need to identify alternative safe and efficacious treatment options for TL.

Thirty years ago, Goad et al, reported an inhibitory effect of terbinafine on cultured promastigotes of the *Leishmania mexicana* complex species [7]. Terbinafine is a member of the allylamine drug group, together with butenafine and naftifine.

Allylamines inhibit squalene -2,3-epoxidase causing accumulation of squalene and depletion of sterols in *Leishmania* amastigotes, resulting in growth inhibition and parasite death[8]. Terbinafine is used as a first line oral treatment for fungal infections, and is the preferred systemic treatment for toenail infections in elderly people for safety reasons [9]. Terbinafine might be a safe option in pregnancy, as no teratogen side effects have been described [10, 11].

Since allylamines might be an attractive alternative TL treatment option, a systematic literature review was performed to assess the efficacy and safety of allylamines in TL treatment and to define priorities for future investigations. All original human, animal and *in vitro* studies, including studies comparing effects of allylamines with placebo or alternative TL treatments, were eligible for inclusion. Cure rate in humans, lesion diameter in animals, promastigote and amastigote viability and growth, and adverse events served as outcome.

Methods

Search strategy

This systematic review, registered in PROSPERO (registration number CRD42018090687, 2018) and available at: https://www.crd.york.ac.uk/prospero/display_record.php?RecordID=90687, followed the Preferred Reporting Items for Systematic Reviews and Meta-analyses (PRISMA) guidelines

A medical information specialist (JL) searched the following electronic databases for studies on leishmaniasis and allylamines, using controlled terms and text words, from inception to May, 24th 2020: MEDLINE (OVID), EMBASE (OVID), the Cochrane Central Register of Controlled Trials (CENTRAL), The Global Health Library, Web of Science, Google Scholar (1st 150 hits) and the clinical trial registers ClinicalTrials.gov and WHO_ICTRP. No language, date or other restriction were applied. The complete search strategies are presented in the Appendix S1. Reference lists and the citing articles of the identified relevant papers were cross-checked in Web of Science for additional relevant studies. The records retrieved were imported and de-duplicated in EndNote.

Study eligibility

All original human, animal and *in vitro* studies were eligible. Eligible studies examined the effects of allylamines with the following endpoints: cure rate in humans, skin lesion diameter in animals and promastigote or amastigote *in vitro* growth or viability in the laboratory. The presence of *Leishmania* parasites had to be confirmed in the study by either microscopy, culture or molecular techniques.

Study selection

JB and JvdE independently screened the identified studies using EndNote and resolved differences through discussion or consultation with a third reviewer (HS). Studies included during title and abstract screening were subsequently assessed as full text. Authors of conference abstracts were contacted to request unpublished data. If the full report was written in another language than English, Spanish, German, Dutch, French or Portuguese, authors were requested to provide a translation.

Data extraction

The following data from all included studies were entered in Excel: study setting, study population, *Leishmania* species, allylamine studied and treatment combinations. From human studies the following information was recorded: age, gender, lesion type and duration, drug presentation, treatment scheme, cure rates, adverse events and information for assessment of risk of bias. Cure rates were calculated according to intention to treat analysis and cure was defined as complete epithelialization of lesions at last available follow up.

From animal studies the following information was recorded: age, gender, lesion type and duration, presentation, treatment scheme, effect on lesion diameter, adverse events and information for assessment of risk of bias.

From *in vitro* studies the following information was recorded: drug concentrations, promastigote or amastigote growth or viability, and culture cytotoxicity.

JB and JvdE extracted data in duplicate and resolved differences trough discussion.

Risk of bias assessment

JB and JvdE independently assessed the quality of the clinical trials, using the revised Cochrane collaborations tool for assessing risk of bias in randomized controlled trials and the Cochrane tool for non-randomised controlled trials [12, 13]. Animal studies were assessed with the SYRCLE´s risk of bias assessment tool [14].

Results

Studies included

The literature search identified 312 unique studies of which 75 were included for full text assessment after screening of titles and abstract. After full text examination, 22 studies were included. Major reasons for exclusion were; `different topic´ and `textbook or review´. The data of two conference abstracts, could not be retrieved by contacting the authors [15, 16], and were therefore excluded from the study. The authors of a Chinese and Farsi study could not provide the data or the English translation of the report and these studies were therefore excluded from the study [17, 18] (Fig 1).

Fig 1. PRISMA literature assessment flow diagram.

Characteristics of the included human trials (n=3), mice studies (n=2), amastigote and promastigote studies (n = 12) are presented in Table 1 - 4. The case reports (n = 5) are presented in Appendix S2.

Table 1: Characteristics of human trials reporting treatment of cutaneous leishmaniasis with terbinafine

First Author	Year	*Leishmania* species	Number of participants	Number of controls	Presentation	Combination
Bahamdan[19]	1997	*tropica*	27	-	systemic	-
Farajzadeh[20]	2016	*tropica*	44	44	topical	meglumine antimoniat
Farajzadeh[21]	2015	*tropica*	40	40	systemic	cryotherapy

[a] Defined as complete epithelialization of lesions at last available follow up and calculated according to intention t

Table 2: Characteristics of mice studies reporting treatment of cutaneous leishmaniasis with terbinafine

First Author	Year	*Leishmania* species	Number of participants	Number of controls	Presentation	Dosis / day	Days treated
Sampaio[22]	2003	*amazonensis*	15	29	systemic	100mg/kg	20
Zakai[23]	2000	*major*	20	40	systemic	0,2mg	28

* no significant difference with terbinafine treated mice

** significant difference with terbinafine treated mice

Table 3: Characteristics of in vitro amastigote studies reporting on effects of allylamines in cutaneous *Leishman*

First Author	Year	*Leishmania* species	Allylamine	Amastigote host cell type
Andrade Neto[24]	2013	*amazonensis*	terbinafine	mice peritoneal macrophages
Berman[25]	1987	*major*	terbinafine	human monocyte derived macrophages
Berman[25]	1987	*major*	naftifine	human monocyte derived macrophages
Bezerra Souza[26]	2016	*amazonensis / braziliensis*	butenafine	mice peritoneal macrophages
Vannier-Santos[27]	1995	*amazonensis*	terbinafine	mice peritoneal macrophages

* combined with 0,001 µM Ketoconazole

IC50: Half maximal inhibitory concentration, ED50: Median effective dose, MIC: Minimum inhibitory concentratio

Table 4: Characteristics of in vitro promastigote studies reporting on effects of allylamines in cutaneous *Leishm*

First Author	Year	***Leishmania* species**	Allylamine	Effective concentration	Unit	Pa
Andrade Neto[24]	2013	*amazonensis*	terbinafine	15	µM	
Andrade Neto[28]	2011	*amazonensis*	terbinafine	8	µM	
Andrade Neto[29]	2009	*amazonensis*	terbinafine	4 - 9	µM	
Beach[30]	1989	*multiple*	terbinafine	27	µM	26-
Bezerra Souza[26]	2016	*amazonensis / braziliensis*	butenafine	34 - 81	µM	
Chance[31]	1999	*amazonensis*	terbinafine	34	µM	>2 fo
Goad[7]	1985	*mexicana*	terbinafine	34	µM	
Rangel[32]	1996	*mexicana / braziliensis*	terbinafine	5 - 15	µM	
Tariq[33]	1994	*tropica*	terbinafine	1373	µM	
Vannier-Santos[27]	1995	*amazonensis*	terbinafine	1	µM	
Zakai[34]	2003	*major*	terbinafine	6	µM	
		mexicana	terbinafine	No inhibition	-	

IC50: Half maximal inhibitory concentration, ED50: Median effective dose, MIC: Minimum inhibitory concentratio

Three human trials assessed the efficacy of terbinafine in humans infected with *L.tropica* [19-21]. One animal study reported the efficacy of terbinafine on *L. amazonensis* and one on *L.major*. Eight *in vitro* studies reported on effectivity of terbinafine in promastigote models and four studied effectivity of allylamines in amastigote models. There were 5 case reports showing a curative effect of terbinafine. In two case reports terbinafine cured a *L. tropica* infected patient [35, 36]. In a HIV positive patient, infected in Colombia and cured with terbinafine, the causative *Leishmania* species was unknown [37]. In another case terbinafine 250mg daily combined with a Crotamiton 10% + Sulphur 2% cream cured a Kenyan patient with cutaneous leishmaniasis, but the causative *Leishmania* species was unknown [38]. Terbinafine combined with itraconazole cured a mucocutaneous and visceral leishmaniasis infection, caused by *L. infantum,* in a patient with liver cirrhosis [39].

Risk of bias assessment

Two randomised controlled clinical trials [20, 21], a one arm non randomised trial [19], and two animal trials [22, 23] were assessed for risk of bias. Farajzadehs randomised controlled trial in 2015 had an acceptable risk of bias [21]. The study of Farajzadeh 2016 lost 73% of patients to follow up [20] and Bahamdans study had severe deviations from intended interventions and 48% loss to follow up [19], leading to an overall judgement of high risk of bias for both. The two mice studies suffered from high risk of bias in various domains including allocation concealment and blinding of outcome assessment (Table 5-7).

Table 5: Risk of bias assessment of included randomised controlled trials in cutaneous leishmaniasis[a]

Study	Randomisation proces	Effect of assignment to intervention	Effect of adhering to intervention	Missing outcome data
Farajzadeh 2016	low	low	some concerns	high
Farajzadeh 2015	low	some concerns	some concerns	low

a The Revised Cochrane risk-of-bias tool for randomized trials (RoB2) was applied for the evaluation

Table 6: Risk of bias assessment of included non-randomised controlled trials in cutaneous leishmaniasis[a]

Study	Confounding	Selection of participants into the study	Classification of interventions	Deviations from intended interventions
Bahamdan 1997	moderate	low	low	critical

a The Risk Of Bias In Non-randomized Studies – of Interventions (ROBINS-I) assessment tool was applied for the ev

Table 7: Risk of bias assessment of included mice studies in cutaneous leishmaniasis[a]

Study	Sequence generation	Baseline characteristics	Allocation concealment	Random housing
Sampaio 2003	low	unclear	high	unclear
Zakai 2000	unclear	high	high	unclear

a SYRCLE's risk of bias tool for animal studies was applied for the evaluation

It was not possible to perform a meta-analysis of the study outcomes, due to the small number of studies, their heterogeneity and low quality.

Efficacy of terbinafine on *L. tropica* human infections and adverse events

The clinical trial of Farajzadeh 2015[21] was the only human *clinical* study with acceptable risk of bias. It included 80 *L. tropica* infected patients randomized between two different treatment groups: 1) oral terbinafine 125-500mg (weight dependent) daily during 4 weeks, combined with cryotherapy every two weeks (n=40) and 2) meglumine antimoniate 15mg/kg/day for 3 weeks combined with cryotherapy every two weeks (n=40). Complete follow up was achieved for all patients at 3 months. Cure rates were calculated according to intention to treat analysis defining cure as complete epithelialization at last available follow up. In the terbinafine arm 15/40 (38%) patients were cured and in the meglumine antimoniate arm 21/40 (53%) cases were cured, but this difference was not statistically significant in Kaplan Meier analysis (p=0,27). None of the *in vivo* studies reported adverse events [19-23].

Species specific effectivity of allylamine treatments

Growth inhibition of terbinafine in promastigote *in vitro* studies was reported for the *L. major, L. tropica, L. mexicana, L. braziliensis and L. guyanensis* complexes. An interspecies comparison in promastigote cultures with terbinafine 27 µM showed higher inhibition levels in old world (*L. major*, *L. tropica* and *L.aethiopica*) species (Table 8).

Table 8: Overview of clinical and in vitro *Leishmania* species specific results of terbinafine in cutaneous leishma[illegible]

Complex	Species	Growth inhibition in promastigotes at 27 µM terbinafine	Effective doses in promastigotes
L.major	*L.major*	52-90%	IC50: 6 µM
L. tropica	*L. tropica*	92-93%	MIC: 1373 µM
	L. aethiopica	90%	-
L. mexicana	*L. mexicana*	26%	no inhibition / MIC: 15 µM / MIC: 34 µ[illegible]
	L. amazonensis	74%	IC50: 4 - 9 µM / MIC: 1 µM
L. braziliensis	*L.braziliensis*	72%	MIC: 1-5 µM
L. guyanensis	*L. guyanensis*	49%	-
	L. panamanensis	41%	-

[a]Defined as complete epithelialization of lesions at last available at follow up and calculated according to intentio[illegible]

[b] combined with 0,001 µM Ketoconazole

IC50: Half maximal inhibitory concentration, ED50: Median effective dose, MIC: Minimum inhibitory concentratio

Treatment with butenafine killed *in vitro* cultured amastigotes of *L. amazonensis* and *L. braziliensis* at a mean effective dose of 30-38µM, compared to the median cytotoxic concentration of 98µM. Naftifine killed *L.major* amastigotes with mean effective dose of 45 µM whilst cytotoxicity levels were more than 110 µM.

Terbinafine drug combination treatment

Various *in vitro* studies evaluated the combination of terbinafine with drugs from the triazole group. An up to 300-fold improvement of the inhibition of *L.braziliensis* promastigotes when combining ketoconazole with terbinafine was demonstrated [32]. Another study reported that ketoconazole and terbinafine had a synergistic effect on the inhibition of *L.amazonensis* amastigotes, resulting in a minimally inhibitory concentration of 0,001µM.

A case of serious mucocutaneous and visceral *L. infantum* leishmaniasis was cured with a daily dose of terbinafine 500mg and itraconazole 200mg, both for 6 months (Table 9).

Table 9: Results of *in vitro* and clinical studies on the combination of Terbinafine with other treatment in tegum

Study	*Leishmania* species	Target	Combined terapy	
Andrade-Neto 2013[24]	*L.amazonensis*	promastigote	LBqT01	
Andrade-Neto 2013	*L.amazonensis*	promastigote	imipramine	
Andrade-Neto 2013	*L.amazonensis*	amastigote	LBqT01	
Andrade-Neto 2013	*L.amazonensis*	amastigote	imipramine	
Vannier Santos 1995[27]	*L. amazonensis*	amastigote	ketoconazole	
Rangel 1996[32]	*L. braziliensis*	promastigote	ketoconazole	
Rangel 1996	*L. braziliensis*	promastigote	D0870	
Vellin 2005[39]	*L.infantum*	human severe mucosal	itraconazole	c
Mawenzi 2018[38]	unknown	human cutaneous	crotamiton + sulphur	c
Farajzadeh 2015[21]	*L. tropica*	human cutaneous	cryotherapy	
Farajzadeh 2016[20]	*L. tropica*	human cutaneous	meglumine antimoniate	

[a]Synergism defined as fractional inhibitory concentration index (FICI) ≤ 0,5

[b]Additive effect defined as: 0,5 < FICI < 4

[c]Synergism defined as total fractional inhibition higher than expected from adding up the fractional inhibition of e
[d]Synergism defined as 300-fold reduction of the Minimum Inhibitory Concentration of ketoconazole with 1µM ter

Discussion

This systematic review comprises an exhaustive search of eight electronic databases and trial registers without language restrictions. It assesses the risk of bias of two randomized controlled trials, a non-controlled trial and two animal studies and summarizes the available evidence including *in vitro* studies and case reports.

Due to limited availability of studies, only 22 primary studies could be included in the systematic review, twelve being *in vitro* studies. Underlying data of two conference abstracts and translation of two papers could not be retrieved. The current review retrieved three human trials on terbinafine for the treatment of TL, but two were not reliable due to high loss to follow up of study cases.

The only well-designed randomised controlled trial of Farajzadeh et al that compared the treatment efficacy of oral terbinafine versus intra muscular meglumine antimoniate showed a non-significant lower cure rate for terbinafine (38% vs 53% of treated patients) [21]. The infecting *Leishmania* species was not confirmed in this study, but is assumed to be *L. tropica* as more than 95% of CL cases in study area (Kerman) are caused by this species be [21, 40].

The comparison of a low sensitivity of amastigotes to allylamines with the clinically achievable levels of terbinafine explains the very low cure rate in *L.tropica*. Table 3 shows that terbinafine, butenafine and naftifine eliminate amastigotes at concentrations between 23 and 45 µM. Faergemann et al studied the terbinafine levels in the dermis and epidermis (where amastigotes

reside in TL) of twelve healthy volunteers during 28 days of daily treatment with 250 mg terbinafine. Mean terbinafine levels were 1,03 µg/g which equals to 3,4 µM [41]. As pharmacokinetics of terbinafine in healthy volunteers are linear between 50 and 750mg [42, 43], the estimated mean terbinafine level of participants in Farajzadeh's study (taking doses between 125 and 500mg daily according to age during 28 days) is 7 µM [21]. Thus, the clinically achieved terbinafine levels were probably five times lower than required and therefore *Leishmania* amastigotes were not eliminated and no effect on cure rate was seen during the clinical trial.

Whilst this review shows that terbinafine is not effective in the treatment of TL, it is highly effective in the treatment of mycotic skin disease. The difference may be due to the high sensitivity to terbinafine of skin fungus compared to *Leishmania* amastigotes. Terbinafine eliminates skin fungus *in vitro* at a mean concentration of 0,014 µM [44], thus is approximately 2500 times more effective than the elimination of *Leishmania* amastigotes.

Promastigote cultures are relatively easy and cheap to perform, but are not very reliable as predictors of *in vivo* effectivity as they represent the infective mosquito stage of the parasite whilst human infection is sustained by intracellular amastigotes [45, 46]. Therefore the results of table 8 which indicates effective concentrations of 1-34µM of terbinafine in *L.braziliensis* and *L.amazonensis* promastigotes should be interpreted with caution. Results of promastigote studies have to be confirmed in amastigote studies.

In 1999, Perez [47] reported the promising use of terbinafine for the treatment of leishmaniasis. His conclusion was based on a previous study by Bahamdan [19] which had critical risk of bias in the present systematic review. Subsequently, Farajzadeh et al [21] has published a study that failed to confirm the efficacy of terbinafine in humans for the treatment of cutaneous leishmaniasis

caused by *L.tropica*. Given the lack of evidence for the efficacy of terbinafine in TL, we cannot confirm expectations raised by Perez.

Allylamines inhibit squalene epoxidase, leading to squalene accumulation and lower sterol levels in promastigotes [8]. Triazole drugs (e.g.: Ketoconazole, Fluconazole and Itraconazole) affect amastigote sterol synthesis by inhibiting C-14α demethylase. A systematic review on triazole therapy for tegumentary leishmaniasis did not show effectivity of monotherapy [48]. Table 3 and 9 show synergistic effects of terbinafine combined with ketoconazole, which might be explained by more effective inhibition when two steps of the same pathway are blocked. Another explanation for the synergism of terbinafine with triazole drugs is that the accumulation of both squalene and 14-methyl sterols is toxic for the amastigotes [32].

Terbinafine combined with ketoconazole eliminated *L.amazonensis* amastigotes at levels of 0,001 µM of both drugs. Terbinafine would reach those levels with an oral dose of 250mg, but the best combination with a triazole drug still has to be defined[41]. Triazole drugs like ketoconazole and fluconazole are inhibitors of the enzymes CYP 2C9 and CYP 3A4, involved in terbinafine metabolism, and may cause significant rise in terbinafine plasma concentrations. Secondary effects of terbinafine combined with triazoles have not been studied extensively and would require large clinical studies before implementation [49, 50].

This review shows the ineffectivity of allylamines as monotherapy for TL. Further trials of allylamines as a treatment for TL should be avoided as the results of a well performed trial were disappointing and *in vitro* studies indicate minimal effective concentrations that are not achieved clinically. The combination of allylamines with triazole drugs in *L.amazonensis* requires more investigation starting with high quality animal studies in order to define optimal doses and safety

profiles and followed by well-designed trials in humans in case of positive findings.

Conclusion

Based on a systematic review of available literature we conclude that allylamine monotherapy is not effective against TL and scarce resources should be invested in other medications. However, the *in vitro* synergistic effects of allylamines combined with triazole drugs against amastigotes, warrants further exploration for the potential clinical effect of allylamine on patients with TL

Supporting information

S1 Appendix. Full electronic search.

S2 Appendix. Characteristics of case reports

S3 Appendix. PRISMA 2009 checklist

References

1. Alvar J, Velez ID, Bern C, Herrero M, Desjeux P, Cano J, et al. Leishmaniasis worldwide and global estimates of its incidence. PLoS ONE. 2012;7(5):e35671.
2. Molyneux DH, Savioli L, Engels D. Neglected tropical diseases: progress towards addressing the chronic pandemic. Lancet (London, England). 2017;389(10066):312-25.
3. Cincura C, De Lima CMF, Machado PRL, Oliveira-Filho J, Glesby MJ, Lessa MM, et al. Mucosal leishmaniasis: A retrospective study of 327 cases from an endemic area of Leishmania (Viannia) braziliensis. American Journal of Tropical Medicine and Hygiene. 2017;97(3):761-6.
4. Hodiamont CJ, Kager PA, Bart A, de Vries HJ, van Thiel PP, Leenstra T, et al. Species-directed therapy for leishmaniasis in returning travellers: a comprehensive guide. PLoS neglected tropical diseases. 2014;8(5):e2832.
5. Oliveira LF, Schubach AO, Martins MM, Passos SL, Oliveira RV, Marzochi MC, et al. Systematic review of the adverse effects of cutaneous leishmaniasis treatment in the New World. Acta tropica. 2011;118(2):87-96.
6. de Vries HJ, Reedijk SH, Schallig HD. Cutaneous leishmaniasis: recent developments in diagnosis and management. American journal of clinical dermatology. 2015;16(2):99-109.
7. Goad LJ, Holz GG, Jr., Beach DH. Effect of the allylamine antifungal drug SF 86-327 on the growth and sterol synthesis of Leishmania mexicana mexicana promastigotes. Biochem Pharmacol. 1985;34(20):3785-8.
8. Petranyi G, Ryder NS, Stutz A. Allylamine derivatives: new class of synthetic antifungal agents inhibiting fungal squalene epoxidase. Science. 1984;224(4654):1239-41.
9. Kreijkamp-Kaspers S, Hawke K, Guo L, Kerin G, Bell-Syer SE, Magin P, et al. Oral antifungal medication for toenail onychomycosis. Cochrane Database Syst Rev. 2017;7:Cd010031.
10. Kaul S, Yadav S, Dogra S. Treatment of Dermatophytosis in Elderly, Children, and Pregnant Women. Indian dermatology online journal. 2017;8(5):310-8.
11. Terbinafine. Drugs and Lactation Database (LactMed). Bethesda (MD): National Library of Medicine (US); 2006.
12. Higgins JPT SJ, Savović J, Page MJ, Hróbjartsson A, Boutron I, Reeves B, Eldridge S. A. A revised tool for assessing risk of bias in randomized trials. Cochrane Methods Cochrane Database of Systematic Reviews 2016(10):(Suppl 1).
13. Sterne JAC, Hernán MA, Reeves BC, Savović J, Berkman ND, Viswanathan M, et al. ROBINS-I: a tool for assessing risk of bias in non-randomized studies of interventions. british journal of medicine. 2016;355(4919).
14. Hooijmans CR, Rovers MM, de Vries RB, Leenaars M, Ritskes-Hoitinga M, Langendam MW. SYRCLE's risk of bias tool for animal studies. BMC medical research methodology. 2014;14:43.
15. Ukşal Ü, Şahin İ, Durgut S, Özcan H, Gürel MS. In vitro and in vivo effect of terbinafine in cutaneous leishmaniasis. Journal of the European Academy of Dermatology and Venereology. 1998;11(Suppl 2):s242.
16. Bonatto AF, Bonatto SF, Sampaio RNR, Vilela H, Vexenat A. Estudo da eficácia da terbinafina, in vitro. Resumos do IV Congresso de Iniciação Científica da Universidade de Brasília 1998. p. 128.
17. Lu-juan GAO, Jin YU, Yaguchi T, Wei C, Ruo-yu LI. A case of cutaneous leishmaniasis misdiagnosed as cutaneous aspergillosis and successfully treated with terbinafine. Chinese Journal of Dermatology. 2011;44(11):811-3.
18. Ebrahimian S, Asilian A, Faghihi G. Comparetaive Study on Glucantime and Oral Terbinafine along with Systemic Glucantime on Cutaneous Leishmaniasis. J Isfahan Med Sch. 2011;28(118):1246-52.

19. Bahamdan KA, Tallab TM, Johargi H, Nourad MM, Ibrahim K, el Sherbini AH, et al. Terbinafine in the treatment of cutaneous leishmaniasis: a pilot study. Int J Dermatol. 1997;36(1):59-60.

20. Farajzadeh S, Heshmatkhah A, Vares B, Mohebbi E, Mohebbi A, Aflatoonian M, et al. Topical terbinafine in the treatment of cutaneous leishmaniasis: triple blind randomized clinical trial. J Parasit Dis. 2016;40(4):1159-64.

21. Farajzadeh S, Esfandiarpour I, Haghdoost AA, Mohammadi S, Mohebbi A, Mohebbi E, et al. Comparison between Combination Therapy of Oral Terbinafine and Cryotherapy versus Systemic Meglumine Antimoniate and Cryotherapy in Cutaneous Leishmaniasis: A Randomized Clinical Trial. Iranian J Parasitol. 2015;10(1):1-8.

22. Sampaio RN, Takano GH, Malacarne AC, Pereira TR, de Magalhaes AV. [In vivo Terbinafine inefficacy on cutaneous leishmaniasis caused by Leishmania (Leishmania) amazonensis in C57BL/6 mice]. Rev Soc Bras Med Trop. 2003;36(4):531-3.

23. Zakai HA, Zimmo SK. Effects of itraconazole and terbinafine on Leishmania major lesions in BALB/c mice. Ann Trop Med Parasitol. 2000;94(8):787-91.

24. Andrade-Neto VV. Papel do colesterol exógeno nos mecanismos de adaptação de Leishmania spp a condições de estresse metabólico e farmacológico. Rio de Janeiro: Fundação Oswaldo Cruz. Instituto Oswaldo Cruz; 2013.

25. Berman JD, Gallalee JV. In vitro antileishmanial activity of inhibitors of steroid biosynthesis and combinations of antileishmanial agents. J Parasitol. 1987;73(3):671-3.

26. Bezerra-Souza A, Yamamoto ES, Laurenti MD, Ribeiro SP, Passero LF. The antifungal compound butenafine eliminates promastigote and amastigote forms of Leishmania (Leishmania) amazonensis and Leishmania (Viannia) braziliensis. Parasitol Int. 2016;65(6 Pt A):702-7.

27. Vannier-Santos MA, Urbina JA, Martiny A, Neves A, de Souza W. Alterations induced by the antifungal compounds ketoconazole and terbinafine in Leishmania. J Eukaryot Microbiol. 1995;42(4):337-46.

28. Andrade-Neto VV, Cicco NN, Cunha-Junior EF, Canto-Cavalheiro MM, Atella GC, Torres-Santos EC. The pharmacological inhibition of sterol biosynthesis in Leishmania is counteracted by enhancement of LDL endocytosis. Acta Trop. 2011;119(2-3):194-8.

29. Andrade-Neto VV. Avaliação da utilização de colesterol exógeno por Leishmania amazonensis como possível alvo farmacológico. Rio de Janeiro: Fundação Oswaldo Cruz. Instituto Oswaldo Cruz; 2009.

30. Beach DH, Goad LJ, Berman JD, Ellenberger TE. Effects of a squalene-2, 3-epoxidase inhibitor on propagation and sterol biosynthesis of Leishmania promastigotes and amastigotes. In: Hart DT, editor. Leishmaniasis. New York, USA: Plenum; 1989.

31. Chance ML, Havercroft PR, Goad LJ. Observations on leucine incorporation into sterol by Leishmania, and its inhibition by terbinafine. Ann Trop Med Parasitol. 1999;93(2):185-8.

32. Rangel H, Dagger F, Hernandez A, Liendo A, Urbina JA. Naturally azole-resistant Leishmania braziliensis promastigotes are rendered susceptible in the presence of terbinafine: comparative study with azole-susceptible Leishmania mexicana promastigotes. Antimicrob Agents Chemother. 1996;40(12):2785-91.

33. Tariq P, Khan KA. Comparative study on susceptibility of Leishmania isolated from dermal leishmaniasis to antifungal drug terbinafine and classic antileishmanial drugs. Pakistan Journal of Pharmacology. 1994;11(1):9-17.

34. Zakai HA, Zimmo S, Fouad MA. Effect of itraconazole and terbinafine on Leishmania promastigotes. J Egypt Soc Parasitol. 2003;33(1):97-107.

35. Scarisbrick JJ, Chiodini PL, Watson J, Moody A, Armstrong M, Lockwood D, et al. Clinical features and diagnosis of 42 travellers with cutaneous leishmaniasis. Travel Medicine and Infectious Disease. 2006;4(1):14-21.

36. Albanese G, Di Cintio R, Galbiati G. Cutaneous leishmaniasis in the north of Italy. Int J Dermatol. 1996;35:223-4.

37. Gonzalez-Ruperez J, Javaloyas de Morlius M, Moreno Carazo A. Remission of localized cutaneous leishmaniasis in a HIV-positive patient using systemic terbinafine. Dermatology. 1997;194(1):85-6.
38. Mawenzi RL. A case of cutaneous leishmaniasis successfully treated with oral terbinafine in Kenya. International Journal of Research in Dermatology. 2018;4(3):433-6.
39. Vellin JF, Russier M, Mougeot G, Kemeny JL, Gilain L. Leishmaniose nasale. Annales d'Otolaryngologie et de Chirurgie Cervico-faciale. 2005;122(2):100-4.
40. Sharifi I, Aflatoonian MR, Fekri AR, Hakimi Parizi M, Aghaei Afshar A, Khosravi A, et al. A comprehensive review of cutaneous leishmaniasis in kerman province, southeastern iran-narrative review article. Iran J Public Health. 2015;44(3):299-307.
41. Faergemann J, Zehender H, Denouel J, Millerioux L. Levels of terbinafine in plasma, stratum corneum, dermis-epidermis (without stratum corneum), sebum, hair and nails during and after 250 mg terbinafine orally once per day for four weeks. Acta dermato-venereologica. 1993;73(4):305-9.
42. Jensen JC. Clinical pharmacokinetics of terbinafine (Lamisil). Clinical and experimental dermatology. 1989;14(2):110-3.
43. Jones TC. Overview of the use of terbinafine (Lamisil) in children. The British journal of dermatology. 1995;132(5):683-9.
44. Leyden J. Pharmacokinetics and pharmacology of terbinafine and itraconazole. Journal of the American Academy of Dermatology. 1998;38(5 Pt 3):S42-7.
45. van den Bogaart E, Schoone GJ, England P, Faber D, Orrling KM, Dujardin JC, et al. Simple colorimetric trypanothione reductase-based assay for high-throughput screening of drugs against Leishmania intracellular amastigotes. Antimicrob Agents Chemother. 2014;58(1):527-35.
46. De Muylder G, Ang KK, Chen S, Arkin MR, Engel JC, McKerrow JH. A screen against Leishmania intracellular amastigotes: comparison to a promastigote screen and identification of a host cell-specific hit. PLoS Negl Trop Dis. 2011;5(7):e1253.
47. Perez A. Terbinafine: broad new spectrum of indications in several subcutaneous and systemic and parasitic diseases. Mycoses. 1999;42 Suppl 2:111-4.
48. Galvao EL, Rabello A, Cota GF. Efficacy of azole therapy for tegumentary leishmaniasis: A systematic review and meta-analysis. PLoS ONE. 2017;12(10):e0186117.
49. Gupta AK, Daigle D, Paquet M. Therapies for onychomycosis a systematic review and network meta-analysis of mycological cure. Journal of the American Podiatric Medical Association. 2015;105(4):357-66.
50. Gupta AK, Versteeg SG, Shear NH. Common drug-drug interactions in antifungal treatments for superficial fungal infections. Expert opinion on drug metabolism & toxicology. 2018;14(4):387-98.

Figure

Click here to access/download;Figure;Fig 1. PRISMA literature assessment flow diagram.tif

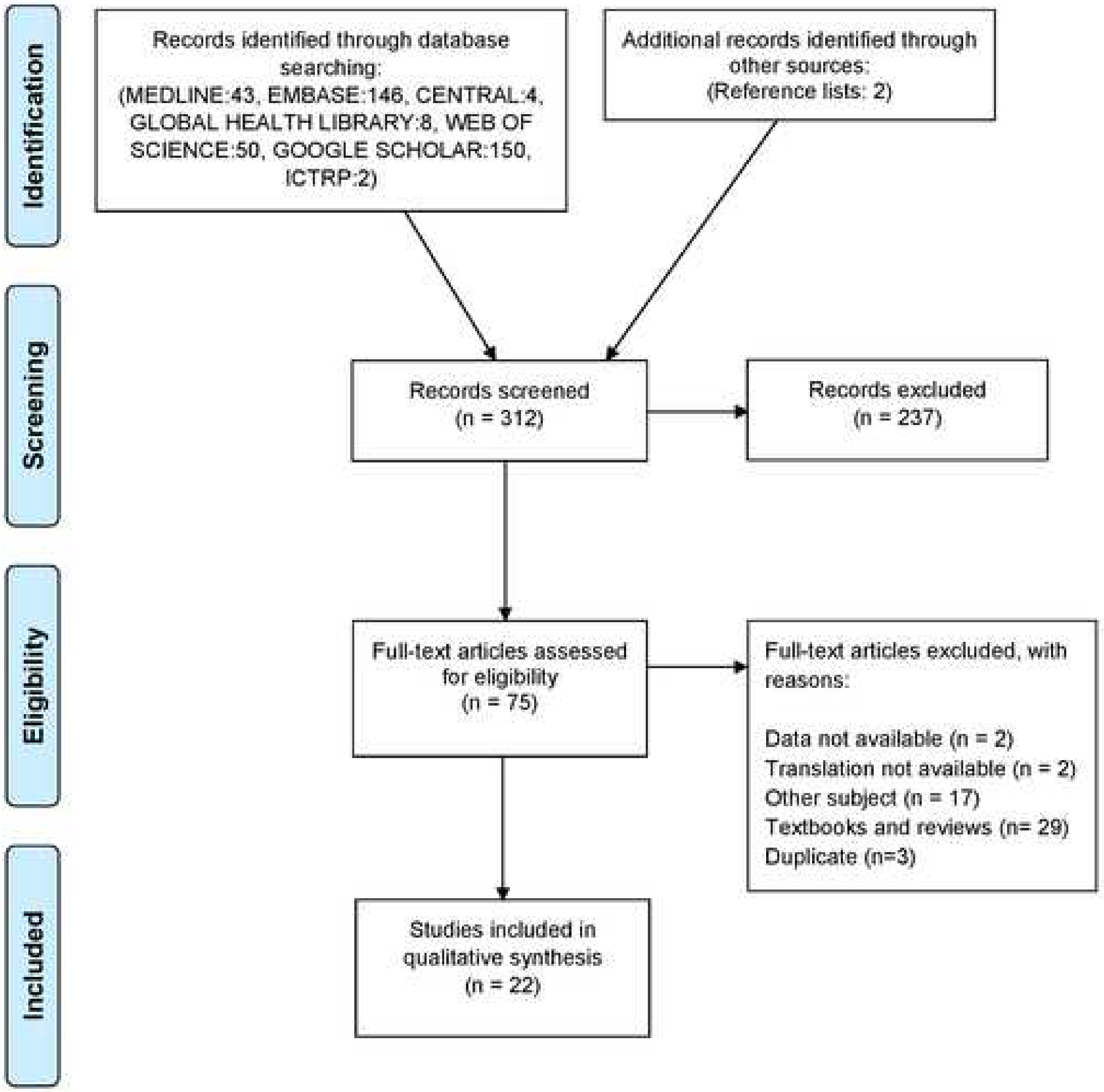

Click here to access/download
Supporting Information
S1 Appendix. Full electronic search.docx

Click here to access/download
Supporting Information
S2 Appendix. Characteristics of case reports (1).docx

Click here to access/download
Supporting Information
S3 Appendix. PRISMA 2009 checklist.docx
