# Supplementary material for: Safety and efficacy of allylamines in the treatment of cutaneous and mucocutaneous leishmaniasis: A systematic review
Source: PLoS One. 2021 Apr 7;16(4):e0249628. doi: 10.1371/journal.pone.0249628 (PMC8026199; doi:10.1371/journal.pone.0249628)
Supplement: Attachment — Submitted filename: Response to Reviewers.docx [file pone.0249628.s005.docx]

Response to Reviewers

Reviewer #1: The methodology of the study seems sound, and the prisma guidelines have been followed. However, I feel the written manuscript needs significant editing both in content and form before it is suitable for publication.

General
-Editing is needed (see pdf for more examples)

*Response: Thank you very much for the revision. The comments in the PDF were applied to the manuscript as applicable. We want to explain that the outcomes are described at the end of the introduction in compliance with the PRISMA checklist. In the discussion we use the comparison with the effectivity of terbinafine in mycotic disease because it is the only generally accepted application of terbinafine in humans.*

• Numbers spelled out or in number (e.g. line 38; one/2)

*Response: Spelling of the numbers was corrected throughout the manuscript.*

• Abbreviations not used consistently

*Response: The use of abbreviations was corrected throughout the manuscript.*

• in vitro is sometimes not in italics

*Response: In vitro was written in italics throughout the manuscript.*

• Tables cannot be viewed properly (last columns have fallen off page), please show in 'landscape orientiation'

*Response: We apologize for the loss of columns during PDF creation and changed the text orientation of the tables that missed columns.*

• Punctuation needs to be checked, sometimes missing or inconsistent, left out wrongly, sometimes overused

*Response: Punctuation was corrected throughout the manuscript.*

-It seems the authors are quite focused on New World leishmaniasis, this should be adjusted if they want to talk about cutaneous and mucocutaneous leishmaniasis in general

*Response: Thanks to your comment, the focus of the written text was adjusted to leishmaniasis in general.*

• the term tegumentary CL is generally used more for the America's and is not common for Old World CL. But the study is also about L. tropica. Perhaps better to talk about cutaneous and mucocutaneous leishmaniasis?

*Response: Thanks to your comment, the term tegumentary was replaced with cutaneous and mucocutaneous throughout the manuscript, including the title.*

• Not all leishmania lesions are necessarily ulcers, in many parts of the world, ulcers are not the dominant presentation

*Response: Thanks to your comment, the text was adapted to include the different presentations of cutaneous leishmaniasis.*

Abstract
-Make abstract more general, instead of mainly New World CL. Also, now it seems almost all mucosal disease is lethal, which is misleading.

*Response: Thanks to your comment the focus of the text was adapted to leishmaniasis in general.*

-Not all current TL treatment have severe side-effects. What about local treatments such as cryotherapy or miltefosine?

*Response: Thanks to your comment, the statement about severe side-effects was adapted.*

Introduction
-Rationale of why a review on this topic is needed should be more clearly explained. Why allylamines and not any other drug? Now the main reason why allylamines are proposed is safety. Its oral form, or low cost (?), availability(?) could be emphasized more

*Response: Thanks to your suggestion the oral form, availability, and cost of terbinafine was mentioned in the introduction.*

-In what capacity do you think allylamines could be used? Once this is defined the introduction can be made more targeted. Now it is explained as an alternative for all CL drugs, which are heaped together as bad and having severe side-effects. But there are plenty of promising treatment initiatives for CL treatment ongoing, thermotherapy, miltefosine.

*Response: Allylamines could potentially be an oral or local, safe, easy to administer, and affordable alternative for the current arsenal of leismananial treatment options, many of whom are either expensive (e.g. miltefosine), painful (e.g. intralesional antimony) or bear severe side effects (e.g. systemic antimony or amphothericine-B)*

• If used as an alternative for systemic pentavalent antimonials (where the major concern is safety) then lesions should also be severe enough to warrant systemic treatment.

*Response: Our objective is a general systematic review that includes all possible cutaneous and mucocutaneous forms. Since the scarcity of data, we did not confine the review to severe forms that warrant systemic therapy only.*

• If intended for more simple lesions, describing the disadvantages of systemic treatment does not make sense.

*Response: Please, see the above written comment.*

• If you want to keep all lesions please explain why other promising safe treatment modalities are not an option.

*Response: Thank you very much for your valuable comment, we added the existence of multiple alternative treatments options in the introduction. The objective of this review is the utility of allylamines for cutaneous and mucocutaneous leishmaniasis. We consider it out of the scope of this paper to analyze the other possible promising safe treatment modalities, as this review compares with the mainstay treatment that’s pentavalent antimonials up to date.*

Methods
-Most studies are not on animals or humans. Any way to assess bias for in vitro studies?

*Response: Although no internationally accepted method for assessing bias in in vitro studies exists, thanks to your comment we applied the method developed by the US National Toxicology Program and implemented the figure in the manuscript.*

Results
-Please also discuss studies with higher risk of bias, now only the 2015 trial is discussed. Just because they have bias does not mean they should be disregarded altogether; it just means they should be interpreted with caution.

*Response: We thank you for your observation and kindly remind you that the results of the studies with high risk of bias can be observed in table 1, which is completely visible now. Additionally, we added a phrase in the methods section in the paragraph `Risk of bias assessment´ stating that studies with a high risk of bias are excluded from the analysis.*

*We feel that it is not wise to discuss the results of studies with high risk of bias, because it might suggest that those results were reliable. We clarified that in the methods section.*

-I would like to see a bit more info on the case reports. Did these patients fail to respond to other treatments? Why were they started on allylamines otherwise?

*Response: Thanks to your comment, the reason to start with terbinafine was stated in the S2 table and in the results section.*

-I would prefer one table with all evidence on effectiveness and one table on characteristics of all studies, separated per study type. The high number of tables make it hard to get a clear overview.

*Response: Although the heterogeneity of the studies did not permit the aggregation of all tables as proposed, we combined several tables and changed the ROB tables into figures, hoping that it will improve the manuscript reading.*

-Please reconsider structure of results, results seem fragmented, some repetition (e.g. MCL/VL case)

*Response: Thanks to your comments, the result section was restructured.*

-Please ensure tables are fully legible before approving pdf submission!

*Response: We apologize for the lost columns and adapted the table orientation.*

Discussion
-Do we have enough evidence to say allylamines do not work? It is important to stress that the quality of evidence found was poor, all human studies were only for L. tropica, while leishmaniasis is a very heterogenous disease.

*Response: Thanks to your comment the conclusion was adapted.*

-The discussion needs more structure.

*Response: Thanks to your comment the discussion was restructured.*

-Discussion can be shortened significantly. Now some parts seem more suited for introduction, others for methods, results. There is no need to refer to tables any more in discussion, this is more for results Some more detailed suggestions can be found below.

*Response: Thanks to your comment the references to tables were eliminated from the discussion.*

• Line 218-228 seems to indicate the dose given to patients was too low (this paragraph can be made more concise). Explain whether it is not an option to increase dosage to reach required levels.

*Response: Thanks to your comment the paragraph was adapted.*

• Line 240-245 explain that based on the clinical papers, terbinafine does not seem promising. I think one paragraph summarizing all evidence and stating that the allylamines are not promising is clearer; this should be the first paragraph of the discussion.

*Response: Thanks to your comment, the first paragraph was adapted.*

• Line 246-248: This is more for introduction, it disrupts the flow of the discussion. Better to leave out details about mechanism of action. "Although triazole monotherapy does not seem effective as for treatment of CL patients, results from in vitro studies indicate terbinafine combined with triazole drugs may be effective through a synergistic effect".

*Response: We applicated your suggestion.*

• Line 254-260: this paragraph can be shortened and combined with the previous

*Response: We applicated your suggestion.*

Reviewer #2: 1. Summary of the research and overall impression
a. The authors delivered a well written systematic review that assessed the evidence available on the efficacy and safety of allylamine treatment for tegumentary leishmaniasis. The search strategy was extensive and inclusive, the methodology accurate and well described, and the conclusions clearly formulated. As a note, please be aware that the tables were not fully available (cut-off on the right side of the text) for reviewing, which might have influenced the comments made by the reviewer.
2. Evidence and examples
a. Major issues
i. Line 229 and 261: ‘this review shows that terbinafine is not effective’ versus line 244: ‘lack of evidence for the efficacy of terbinafine’. The authors base this conclusion primarily on one well-designed human trial, together with (indirect) arguments coming from in vitro studies (although this impression might be linked to incomplete availability of the data from the tables). From the text, it seems more correct to state that there is no proof of efficacy, rather than state that there is proof of inefficacy.

*Response: Thank you very much for the revision and comments. The suggestion was applied.*

b. Minor issues
i. Abstract
1. Line 71: ‘Furthermore, poor treatment responses exist for TL drugs’. Please specify: is this so in general/for the majority of patients? Or specifically so for pentavalent antimonials,…? How does this sentence add to line 66 ‘… each species responds differently to treatment’?

*Response: Thanks to your suggestion the sentence was changed.*

2. Line 78: add space between ‘death[8]’

*Response: Thanks for the comment, it was applied.*

3. Line 84: ‘All original human, animal and in vitro studies, including studies comparing effects of allylamines with placebo or alternative TL treatments, were eligible for inclusion’. This sentence seems strangely formulated. Alternative suggestion: ‘all original human, animal and in vitro studies concerning allylamines and leishmaniasis were eligible for inclusion. Comparators – if any - included both placebo or alternative TL treatments’ ?

*Response: Thank you very much for the suggestion, it was applied.*

ii. Methods
1. Line 92: add period at the end of the line.

*Response: Thanks for the comment, it was applied.*

2. Line 97: ‘no language, date or other restriction were applied’. Replace: either ‘other restrictionS were applied’ or ‘other restriction WAS applied’.

*Response: Thanks for the comment, it was applied.*

3. Line 125: ‘presentation’. Add ‘drug’ for clarity (as was done for human studies in line 120).

*Response: Thanks for the comment, it was applied.*

iii. Results
1. Line 175: add space between ‘2015[21]’.

*Response: Thanks for the comment, it was applied.*

2. In the tables with Characteristics of trials (Table 1-4) it is not registered systematically whether or not the treatment was allylamine monotherapy or combination treatment. For human trials and case reports, a combination column is present, but this is not the case for mice studies (Table 2) or in vitro studies. I would suggest the authors to include this column for all tables on characteristics. At present it seems from the table 3 and 4 that in vitro studies all studied allylamine monotherapy, while it only becomes clear in line 194 that most tested combination therapies. [Since Tables 1-4 are not fully visible in the manuscript provided, it is possible that this column is indeed present in all tables, in which case the authors can ignore this comment.]

*Response: Thank you very much for the comment. We added the column indicating combination treatments to the tables with characteristics of trials and in vitro studies (Table 1 and 2 after fusion).*

3. Line 196: add space to ‘L.braziliensis’. Similar mistake on line 198, 219, 237, 238, 244, 254, 264

*Response: Thanks for the comment, it was applied.*

iv. Discussion
1. Line 218: sentence is difficult to understand, try to rephrase? E.g. ‘A low sensitivity of amastigotes to the clinically achievable levels of terbinafine…’?

*Response: Thanks for the comment, it was applied.*

2. Line 256: add space to ‘defined[41]’

*Response: Thanks for the comment, it was applied.*

Reviewer #3: 
The aim of this review article is to assess the safety and efficacy of allylamines in the treatment of tegumentary leishmaniasis. In this search, 22 publications were included, PRISMA statement for reporting was used and also risk of bias in human and animal studies was assessed with the Cochrane and SYRCLE´s tools, respectively.
Overall, the study is interesting and novel; however, the following concerns need to be corrected:

Abstract:
- The conclusion section is brief; please explain more about the message you want to present.

*Response: Thanks to your comment, we extended the conclusion section of the abstract.*

- You shouldn’t sort out the abstract, please check the instruction of the journal.

*Response: We corrected the headings in the abstract as you suggest.*

Introduction:
- Line 61: please delete “Background”.

*Response: Thanks for the comment, it was applied.*

Methods:
- Line 130: “Risk of bias”, please explain more about this section.

*Response: Thanks to your comment, the Risk of bias section was extended.*

Results:
- Tables 1,2,3,4,8: I couldn't see some columns of these tables, please make the tables in the form of landscape or smaller.

*Response: We apologize for the loss of columns during the transformation to PDF. It was corrected.*

- Line 153: After “One animal study reported the efficacy of terbinafine on L. amazonensis and the other on L. major” please mention the references.

*Response: The phrase was removed from the manuscript in response to other comments.*

- Line 155: After “allylamines in amastigote models” mention the references.

*Response: The phrase was removed from the manuscript in response to other comments.*

- Line 183: In the study of Farajzadeh et al., 2015, I found that oral terbinafine may have approximately the same efficacy as glucantime. So please explain more about the finding and conclusion of the study in both discussion and result sections.

*Response: Thanks to your comment we mention the difference in endpoint between Farajzadeh and our systematic review.*

- Line 191: After “compared to the median cytotoxic concentration of 98μ” please mention the references.

*Response: Thanks for the comment, it was applied.*

- Line 193: After “were more than 110 μM” please mention the references.

*Response: Thanks for the comment, it was applied.*

- Line 199: After “resulting in a minimally inhibitory concentration of 0,001μM.” please mention the references.

*Response: Thanks for the comment, it was applied.*

Discussion:
- Line 228: As I said, in the study of Farajzadeh et al., 2015; oral terbinafine demonstrates the same efficacy as glucantime. it seems that in some cases, oral terbinafine could be used for treatment of cutaneous leishmaniasis. So you should explain more about the finding of that study in the discussion.

*Response: Thanks to your comment we explain that the same efficacy was possibly caused by resistance to meglumine antimoniate.*

Conclusion:
- The conclusion section is brief; explain more about the message you want to deliver.

*Response: Thanks to your comment we extended the conclusion section.*
