# Supplementary material for: Safety and efficacy of allylamines in the treatment of cutaneous and mucocutaneous leishmaniasis: A systematic review
Source: PLoS One. 2021 Apr 7;16(4):e0249628. doi: 10.1371/journal.pone.0249628 (PMC8026199; doi:10.1371/journal.pone.0249628)
Supplement: Attachment — Submitted filename: Response to Reviewers.docx [file pone.0249628.s006.docx]

Response to Reviewers

Apart from a few minor issues, I would like to invite you to consider the suggestion to either include the studies at high risk of bias or to make a better case for excluding them (maybe based on study design? see below).

*Response: Thank you very much for the revision. The studies at high risk of bias were not excluded from the systematic review, as explained below, and the phrases “Studies were excluded from analysis if high risk of bias was perceived during assessment” were eliminated from the abstract and methods.*

Journal Requirements:

Please review your reference list to ensure that it is complete and correct. If you have cited papers that have been retracted, please include the rationale for doing so in the manuscript text, or remove these references and replace them with relevant current references. Any changes to the reference list should be mentioned in the rebuttal letter that accompanies your revised manuscript. If you need to cite a retracted article, indicate the article’s retracted status in the References list and also include a citation and full reference for the retraction notice.

*Response: The reference list was checked for retractions with the `scite reference check´ online tool, but none was detected.*

Additional Editor Comments:

- Abstract and methods: “Studies were excluded from analysis if high risk of bias was perceived during assessment”. How many studies were excluded in this way? This is an unusual approach, especially because you include studies with diverse designs. Unless you have good reasons to do otherwise, I would prefer that you use “study design” at the level of study selection, and report the “risk of bias assessment” as part of the findings (not as an exclusion criterion) and in the discussion. Within the findings of the review, you can then highlight that some studies had high risk of bias (explain the reasons) and that the findings of these studies should be interpreted with caution.

*Response: Thank you for the important comment. We recognize that the phrases about exclusion of studies at high risk of bias in the abstract and methods generated confusion as it was not our intention to exclude them from the entire review as can be seen in Table 1. Therefore, the two phrases were eliminated (line 31 and line 126 in the Revised Manuscript with track changes) and an additional comment was written in the discussion (line 243-246 in the Revised Manuscript with track changes).*

Abstract: terbinafine is safe during pregnancy. It is not only about safety during pregnancy? Consider rephrasing.

*Response: Thanks to your comment, the phrase was adapted to: `Allylamine drugs, like terbinafine, are safe, including during pregnancy. ´*

- Introduction: “antimonial therapy is painful”. Please revise this sentence; I assume it is only painful when applied in the lesion.

*Response: Thanks for your comment, but we prefer to maintain the sentence as antimonial therapy is either applied intra-lesional, intra-muscular, or intra-venously. We consider all three methods to be painful as they require injections.*

- Pentavalent antimonials are the most frequently used drugs for new world leishmaniasis. Is that also true for old world leishmaniasis? See for example [https://apps.who.int/iris/bitstream/handle/10665/44412/WHO_TRS_949_eng.pdf?sequence=1&isAllowed=y](https://eur04.safelinks.protection.outlook.com/?url=https%3A%2F%2Fapps.who.int%2Firis%2Fbitstream%2Fhandle%2F10665%2F44412%2FWHO_TRS_949_eng.pdf%3Fsequence%3D1%26isAllowed%3Dy&data=04%7C01%7Cj.m.bezemer%40amsterdamumc.nl%7C05ec2b859fb043b2f46a08d8de3820f5%7C68dfab1a11bb4cc6beb528d756984fb6%7C0%7C1%7C637503679969598572%7CUnknown%7CTWFpbGZsb3d8eyJWIjoiMC4wLjAwMDAiLCJQIjoiV2luMzIiLCJBTiI6Ik1haWwiLCJXVCI6Mn0%3D%7C2000&sdata=FaIs9o%2BOncdWaSLynNDC7aC3Ji4PI1XBjwUJ9FCuzaA%3D&reserved=0) (Box 2, page 69).

*Response: Thanks to your comment we adapted the statement for old world leishmaniasis to: ` are still the most frequently used for American CL and MCL [5]* ***and frequently*** *used for old world leishmaniasis [6].´(line 53 in the Revised Manuscript with track changes)*

- Methods, study eligibility: check spelling of “species”. The singular is species, not “specie”.

*Response: Thanks to your comment, the spelling of species was corrected*

6. Review Comments to the Author

Reviewer #2: minor suggestions:
- Line 35: remove comma after Leishmania tropica

*Response: Thanks to your comment, we removed the comma.*

- Line 51: ‘… are still the most frequently used ones’ ?

*Response: Thanks to your comment we adapted the statement for old world leishmaniasis.*

- Line 139 + 261: et al. (dot behind al)

*Response: Thanks to your comment, we added the dots behind al.*

- Line 250 : to propose it as (rather than propose it for)?

*Response: Thanks to your comment, the phrase was adapted.*

- Numbers are sometimes written in words, sometimes in numbers; this is not done coherently. Rule of thumb: numbers ten or below should be written out in words; above ten should be written as numbers

*Response: Thanks for your comment. We corrected a number 20 and a number 12 that were written out in words in the first paragraph of the introduction and of the discussion.*
